# Retraction: RNAi-mediated TCF-3 gene silencing inhibits proliferation of Eca-109 esophageal cancer cells by inducing apoptosis

**DOI:** 10.1042/BSR-20170799_RET

**Published:** 2021-09-13

**Keywords:** Cell cycle, Eca-109 cell, Esophageal cancer, SiRNA, Transcription factor 3 gene

This article is being retracted from *Bioscience Reports* at the request of the Editor-in-Chief and the Editorial Board following receipt of a notification from a reader, alerting the Editorial Board to the GAPDH Western blots in Figure 7C which are duplicated in several other publications by different authors. In addition, the Figure 4A NC panel is duplicated in another publication by different authors.

The authors have been contacted in regards to the Retraction, and have not responded to the Journal's queries and the concerns raised. Given the extent of the issues raised, the Editorial Board stand by the decision to retract the article

